# Supplementary material for: The Use of Thioflavin T for the Estimation and Measurement of the Plasma Membrane Electric Potential Difference in Different Yeast Strains
Source: J Fungi (Basel). 2023 Sep 20;9(9):948. doi: 10.3390/jof9090948 (PMC10532974; doi:10.3390/jof9090948)
Supplement: Supplementary file 1 [file jof-09-00948-s001.zip › jof-2566679-supplementary.pdf]

Supplementary Figure S1A.

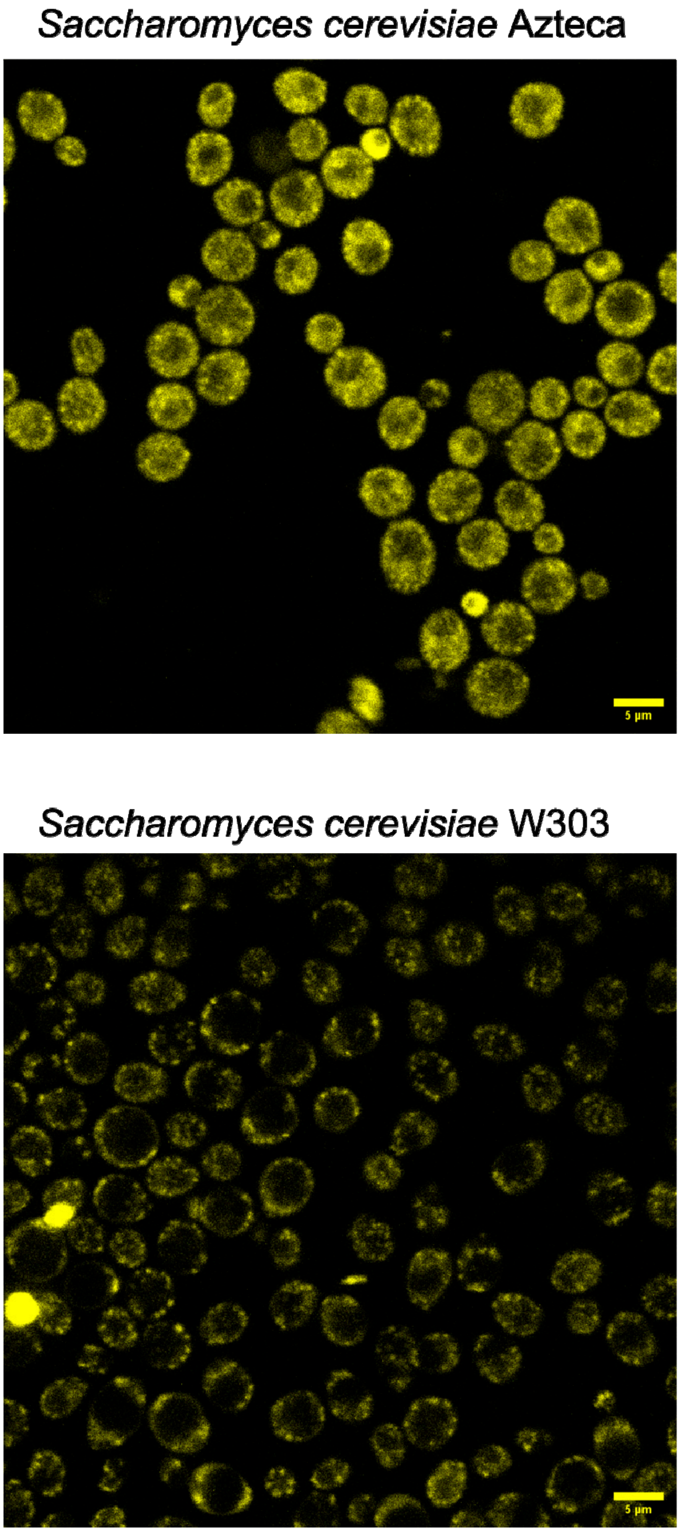

Figure S1. A. Control images enlargement of the different strains of Figure 5.

Supplementary Figure S1B.

Candida albicans

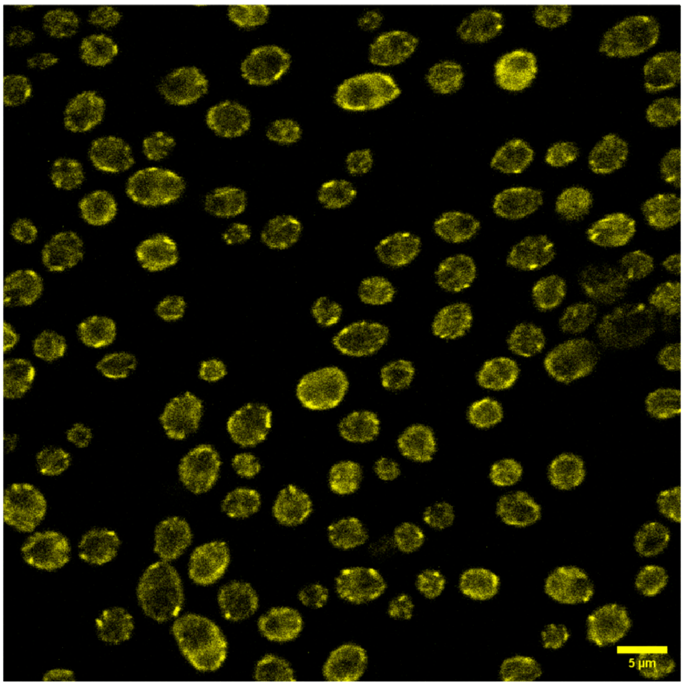

Debaryomyces hansenii

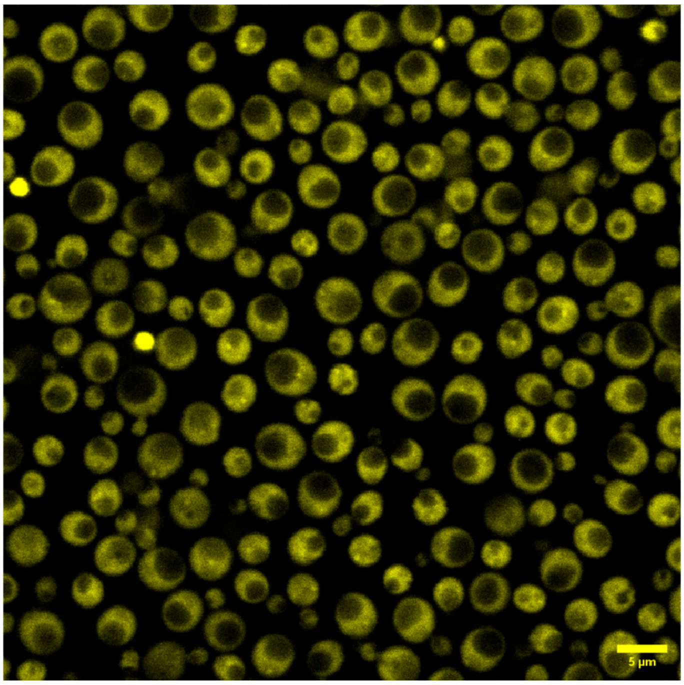

Figure S1. B. Control images enlargement of the different strains of Figure 5.

Supplementary Figure S1C.

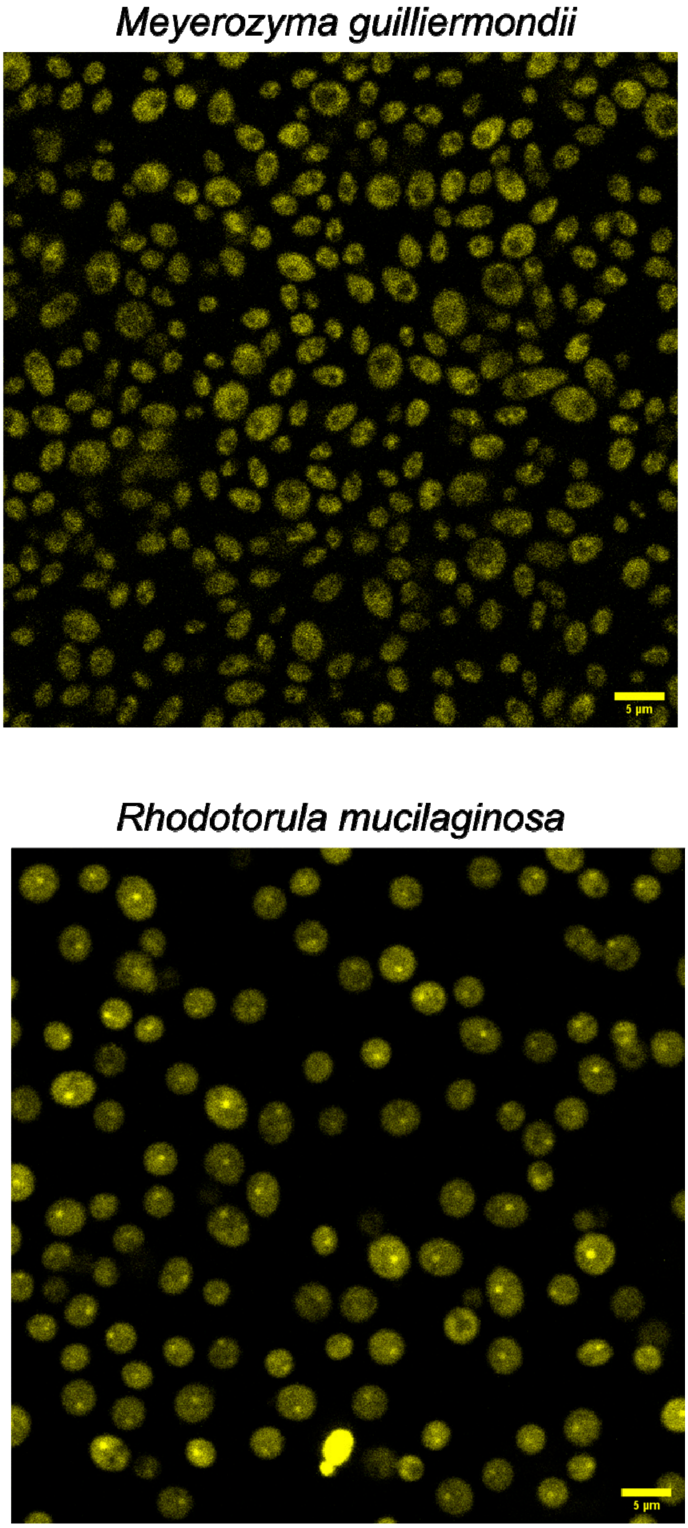

Figure S1. C. Control images enlargement of the different strains of Figure 5.

Supplementary Figure S2.

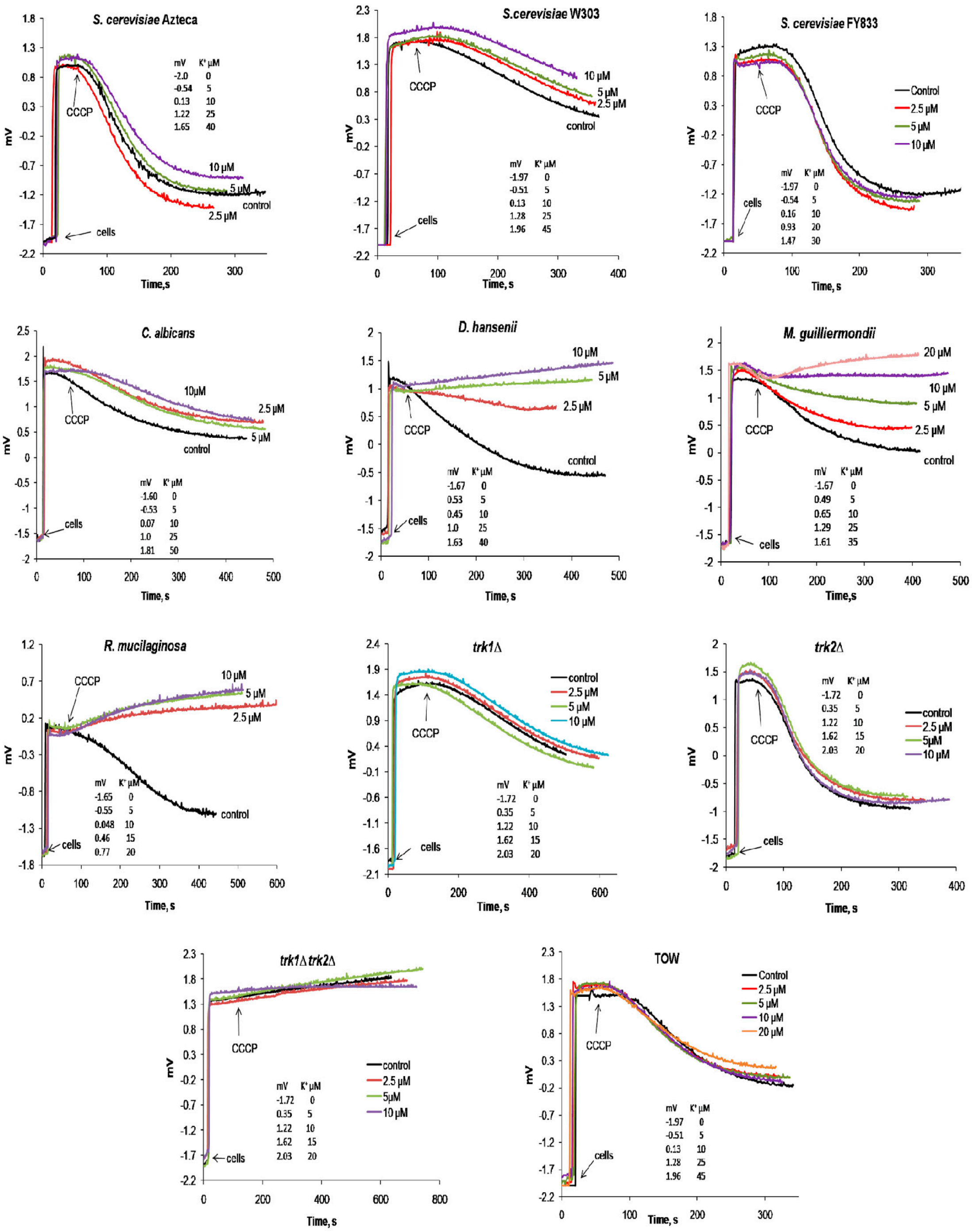

Figure S2. Effects of different CCCP concentrations on the K^+ uptake. The medium used was 2 mM MES-TEA buffer, pH 6.0, 20 mM glucose. Tracings were started by the addition of 100 mg of cells (w. w.). Where indicated, variable concentrations of CCCP were added. A standard curve for the tracings was performed by the successive addition of 0 to 50 µM (specified in the insert).

Supplementary Figure S3

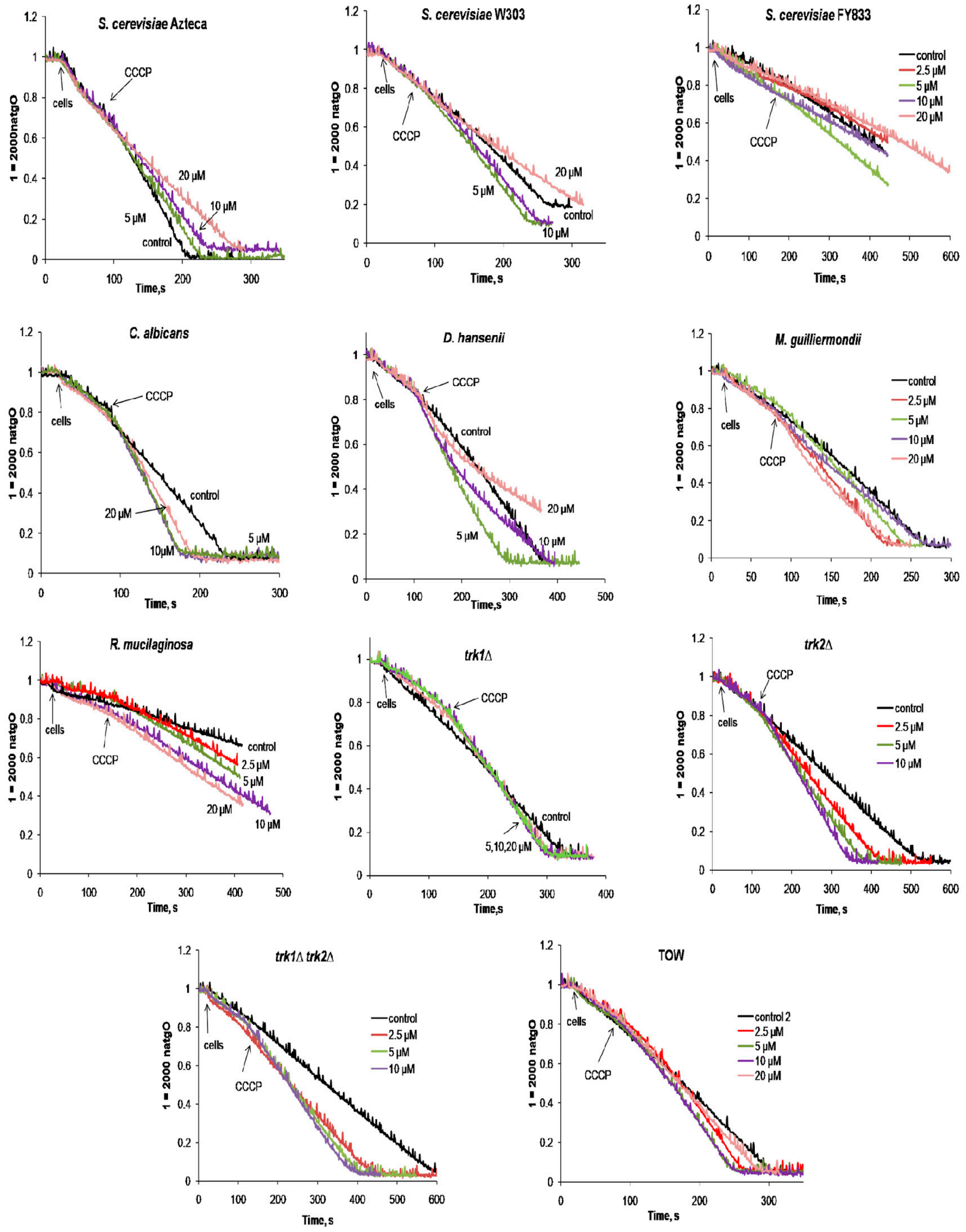

Figure S3. Effects of different CCCP concentrations on oxygen consumption. The medium used was 10 mM MES-TEA buffer, pH 6.0, 20 mM glucose. Tracings were started by the addition of 50 mg of cells (w. w.). Where indicated, variable concentrations of CCCP were added.
